# Creating multithemed ecological regions for macroscale ecology: Testing a flexible, repeatable, and accessible clustering method

**DOI:** 10.1002/ece3.2884

**Published:** 2017-03-26

**Authors:** Kendra Spence Cheruvelil, Shuai Yuan, Katherine E. Webster, Pang‐Ning Tan, Jean‐François Lapierre, Sarah M. Collins, C. Emi Fergus, Caren E. Scott, Emily Norton Henry, Patricia A. Soranno, Christopher T. Filstrup, Tyler Wagner

**Affiliations:** ^1^ Department of Fisheries and Wildlife & Lyman Briggs CollegeMichigan State UniversityEast LansingMIUSA; ^2^ Department of Computer Science & EngineeringMichigan State UniversityEast LansingMIUSA; ^3^ School of Natural SciencesTrinity College DublinDublin, 2Ireland; ^4^ Département de Sciences BiologiquesUniversité de Montréal, Pavillon Marie‐VictorinMontréalQCCanada; ^5^ Center for LimnologyUniversity of WisconsinMadisonWIUSA; ^6^ Department of Fisheries and WildlifeMichigan State UniversityEast LansingMIUSA; ^7^ National Ecological Observatory NetworkBoulderCOUSA; ^8^ Division of Outreach and EngagementOregon State UniversityCorvallisORUSA; ^9^ Large Lakes Observatory & Minnesota Sea GrantUniversity of Minnesota DuluthDuluthMNUSA; ^10^ U.S. Geological SurveyPennsylvania Cooperative Fish & Wildlife Research UnitPennsylvania State UniversityUniversity ParkPAUSA

**Keywords:** constrained spectral clustering, ecoregions, geospatial variables, lake, landscape, macroecology, macrosystems, regional spatial scale, regionalization, spatial heterogeneity

## Abstract

Understanding broad‐scale ecological patterns and processes often involves accounting for regional‐scale heterogeneity. A common way to do so is to include ecological regions in sampling schemes and empirical models. However, most existing ecological regions were developed for specific purposes, using a limited set of geospatial features and irreproducible methods. Our study purpose was to: (1) describe a method that takes advantage of recent computational advances and increased availability of regional and global data sets to create customizable and reproducible ecological regions, (2) make this algorithm available for use and modification by others studying different ecosystems, variables of interest, study extents, and macroscale ecology research questions, and (3) demonstrate the power of this approach for the research question—How well do these regions capture regional‐scale variation in lake water quality? To achieve our purpose we: (1) used a spatially constrained spectral clustering algorithm that balances geospatial homogeneity and region contiguity to create ecological regions using multiple terrestrial, climatic, and freshwater geospatial data for 17 northeastern U.S. states (~1,800,000 km^2^); (2) identified which of the 52 geospatial features were most influential in creating the resulting 100 regions; and (3) tested the ability of these ecological regions to capture regional variation in water nutrients and clarity for ~6,000 lakes. We found that: (1) a combination of terrestrial, climatic, and freshwater geospatial features influenced region creation, suggesting that the oft‐ignored freshwater landscape provides novel information on landscape variability not captured by traditionally used climate and terrestrial metrics; and (2) the delineated regions captured macroscale heterogeneity in ecosystem properties not included in region delineation—approximately 40% of the variation in total phosphorus and water clarity among lakes was at the regional scale. Our results demonstrate the usefulness of this method for creating customizable and reproducible regions for research and management applications.

## 1 Introduction

Ecologists are increasingly conducting research at broad spatial and temporal scales to understand and predict ecosystem responses to environmental pressures such as land use intensification and global climate change. The discipline of macrosystems ecology meets these challenges by considering diverse ecological phenomena at scales of regions to continents (i.e., macroscales) and their interactions with phenomena at other finer scales (Heffernan et al., 2014). Translating fine‐scaled understanding to macroscales is difficult because ecosystems are complex, heterogeneous, and strongly influenced by multiscaled environmental and anthropogenic factors (i.e., ecological context). For example, the importance of local drivers of ecosystem properties and their interactions are well known; yet, there are many examples of regional drivers constraining biological and biogeochemical patterns and processes at local scales (e.g., Bell et al., 1993; Iannone et al., 2015; Reyer et al., 2015; Sobek, Tranvik, Prairie, Kortelainen, & Cole, 2007). Therefore, spatial heterogeneity among ecosystems is a result of complex relationships within and across multiple spatial scales, and the *regional scale* (i.e., intermediate between local and continental scales) provides a vital link for understanding multiscaled ecological phenomena.

One way for ecologists to include the regional scale in their research is to apply a regionalization framework that classifies the landscape into ecological regions. This approach has a long history in geography and biogeography (e.g., Christian, 1958; Whittaker, 1956), with many regionalization frameworks in use globally (e.g., Abell et al., 2008; Bailey, Avers, King, & McNab, 1994; Klijn, De Waal, & Oude Voshaar, 1995; Marshall, Smith, & Selby, 1987). These contiguous regions are used under the assumption that ecosystems within regions are more similar (in properties and in responding to stressors) than those across regions (Seelbach, Wiley, Baker, & Wehrly, 2006). Studies that have included ecological regions have improved scientific understanding of the patterns and processes occurring within and among regions. For example, multiscaled studies have documented that some of the spatial heterogeneity in lake characteristics across broad spatial extents is at the regional scale (i.e., there is a significant amount of among‐region variation in lake nutrients), but that the magnitude of variation attributed to regions depends upon the response variable of interest, the ecological regions used, and the spatial extent of the study (Cheruvelil, Soranno, Bremigan, Wagner, & Martin, 2008; Cheruvelil, Soranno, Webster, & Bremigan, 2013; Jenerette, Lee, Waller, & Carlson, 2002). Studies of lake water quality that have included ecological regions have also quantified complex interactions among landscape features at different spatial scales including differences in both the strength and direction of relationships between lake response variables and local drivers, depending on the region (Fergus, Cheruvelil, Soranno, & Bremigan, 2011; Filstrup et al., 2014; Lottig et al., 2014). The fundamental importance of ecological regions for capturing regional‐scale ecosystem heterogeneity has been recognized beyond the scientific community—they are incorporated into big‐science observatories (e.g., US‐NEON program) and broad‐scale ecosystem assessments (e.g., EU Water Framework Directive, and US EPA National Assessments). In fact, not accounting for regional differences when studying and managing ecosystem responses to environmental drivers could lead to inappropriate or inadequate interpretations and management decisions.

Despite their frequent use, existing ecological regions have characteristics that potentially limit their general application. They were created for specific purposes, each using different underlying data and methods (Cheruvelil et al., 2013), making them unlikely to meet the requirements for research questions outside of those they were originally developed to address (Loveland & Merchant, 2004; McMahon et al., 2001; Thompson et al., 2005). Due mainly to limitations in the availability of broad‐scale geospatial data, most past efforts were based on a relatively small number of terrestrial and climatic characteristics that were quantified at continental or global extents. Many ecological regions were also developed subjectively using paper maps, leading to regions that cannot be reproduced or easily modified for new purposes (Hargrove & Hoffman, 2004). These previously recognized limitations have led to calls for a stronger scientific basis for development of ecological regions for both research and application (McMahon, Wiken, & Gauthier, 2004).

Interestingly, historic regionalization frameworks are widely used for science and applications, despite recent computational advances and increases in the quality and resolution of satellite and map‐based data that provide high‐resolution, continental‐scaled datasets for a wide range of atmospheric, climatic, terrestrial, and freshwater characteristics. Furthermore, there have been advances in statistical and computational approaches for delineating objective and reproducible ecological regions (e.g., Hargrove & Hoffman, 1999; Nguyen, Epps, & Bailey, 2010; Stepinski, Niesterowicz, & Jasiewicz, 2015). However, most of these newer methods have not been broadly disseminated to or available in a form easily adoptable by the ecological community. Additionally, because many of these methods are optimized to maximize landscape homogeneity, they do not always create contiguous regions (Duque, Ramos, & Suriach, 2007; Olden, Kennard, & Pusey, 2012; Yuan, Tan, Cheruvelil, Collins, & Soranno, 2015). Region contiguity is useful for two important reasons. First, such regions help account for broad‐scale spatial autocorrelation that is common among ecosystems (Fortin & Dale, 2005). Second, contiguous regions are useful for management because they allow managers to apply similar practices to nearby but unstudied ecosystems. Therefore, we need methods that create contiguous and homogeneous regions, as well as dissemination of these approaches to the ecological community.

To help fill the need for adaptable and flexible methods for creating regions, we apply a newly published computer science clustering algorithm that creates customized ecological regions, test its use for macrosystems ecology research, and make it available in an online repository. This algorithm, known as “spatially constrained spectral clustering,” is a flexible method that allows users to impose restrictions on whether spatially adjacent points should be in the same region, thereby influencing the clustering process to create homogeneous regions that are also geographically connected (i.e., contiguous). This method was developed and tested using terrestrial landscape data for three U.S. states and was found to outperform three other algorithms for delineating ecological regions (Yuan et al., 2015). Here, we expand on this previous work to: (1) apply the spatially constrained spectral clustering algorithm (Yuan et al., 2015) to create ecological regions with a wider range of nationally available terrestrial, climatic, and freshwater geospatial data for 17 northeastern U.S. states (approximately 1,800,000 km^2^); (2) examine which of the 52 geospatial features were most influential in creating these regions to determine how important individual geophysical features are for ecological region delineation; and (3) test the ability of the resulting 100 ecological regions to capture regional variation in lake characteristics that were not used to develop the regions, i.e., water nutrients and clarity, for ~6,000 lakes. We make this algorithm freely available with an accessible user interface for other researchers to use and modify, including the ability to: create different numbers/sizes of regions; use a subset of themes or different combinations of measures of the terrestrial, atmospheric, and freshwater landscapes; and create regions for a different spatial extent (e.g., state, nation, and continent). This objective and reproducible method and available code for creating ecological regions are designed to support a wide range of macroscale ecology applications.

## 2 Materials and Methods

### 2.1 Study extent and data

**Figure 1:**
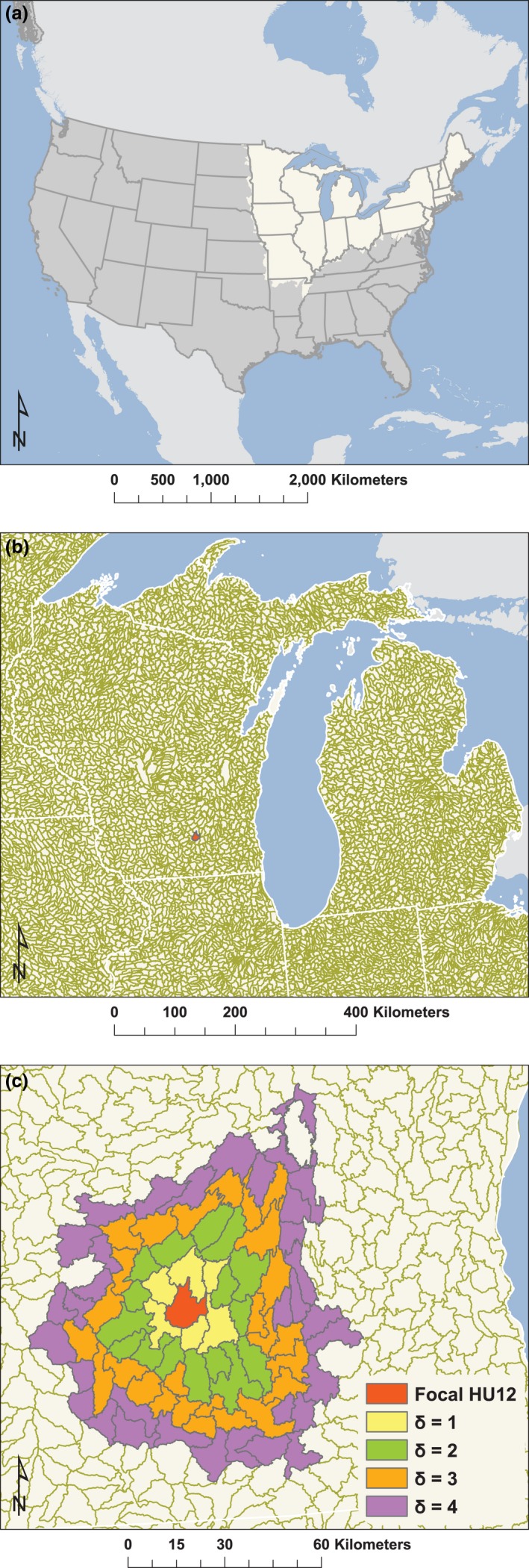
The study extent: (a) The U.S. with the 17 states shaded, (b) A close‐up of HU‐12s in ~2 states (Wisconsin and Michigan), and (c) A close‐up of a focal HU‐12 (red in (b)) with its neighboring HU‐12s shaded to demonstrate different levels of the contiguity constraint

This study uses a harmonized geospatial dataset of landscape and lake ecosystem features called LAGOS‐NE (LAke multiscaled GeOSpatial and temporal database; Soranno et al., 2015). LAGOS‐NE was developed at the subcontinental extent of a land area of ~1,800,000 km^2^ within 17 northeastern U.S. states that have ~50,000 lakes with surface area ≥4 ha (Figure 1). LAGOS‐NE comprises two modules. First, LAGOS‐NE_GEO_ v1.03 includes geospatial data on features including climate, atmospheric deposition, land use and land cover, hydrology, freshwater connectivity, geology, and topography measured across a range of spatial and temporal extents. The base geographic unit used to create ecological regions was the U.S. Geological Survey 12‐digit hydrologic unit (HU‐12), which is based on river basins (Seaber, Kapinos, & Knapp, 1987). There are 20,257 HU‐12s in the study extent, ranging in land area from 0.35 to 1,276 km^2^ (Table 1, Figure 1). The 52 natural geographic variables used in this study were quantified at the HU‐12‐scale in LAGOS‐NE_GEO_ and grouped into three themes: terrestrial landscape features, climate features, and freshwater landscape features (Appendix S1 in Supporting Information). In‐lake or lake‐specific characteristics were not used when creating the regions.

**Table 1:** Descriptive statistics for the hydrologic units (HU‐12s; Seaber et al., 1987) clustered to make regions and the lake characteristics used to test region ability to capture macroscale variation among ecosystems (using means). In‐lake data were from summer samples of lakes ≥4 ha in size during 2002–2011. Water clarity was measured as Secchi disk depth

Variable	Unit	Median	Mean	25th percentile	75th percentile	Sample size
HU‐12	ha	7,868	8,446	5,710	10,580	20,257
Water clarity	m	2.6	2.9	1.4	3.9	6,044
Total Phosphorus	μg/L	16.2	35.2	10.0	32.2	3,896

The second LAGOS‐NE module, LAGOS‐NE_LIMNO_ v1.054.1, includes lake‐specific water quality and chemistry data compiled from 54 individual datasets for a subset of ~10,000 lakes in the study extent (Soranno et al., 2015). For independently testing the ecological regions created in this study (see below for analytical approach), we used summer values for total phosphorus from the surface waters of lakes and for water clarity of the lake measured as the Secchi depth reading. We used these two lake‐specific response variables because they: (1) are important variables for lake functioning and (2) are routinely measured in monitoring programs and thus applicable to regional management (Appendix S2 in Supporting Information). Because lake nutrients and water clarity can vary seasonally and temporally, we used mean summer values (i.e., June 15‐August 15) from the most recent 10 years of data available (i.e., 2002–2011) (Table 1). The data and metadata for all analyses are available in a data repository (Cheruvelil et al., 2016).

### 2.2 Creating ecological regions

#### 2.2.1 Spatially constrained spectral clustering

We created ecological regions using a constrained spectral clustering method recently proposed by Yuan et al. (2015). This objective computational approach is repeatable, allows users to specify multitheme inputs and the number of regions, and takes into account both landscape homogeneity and region contiguity. Yuan et al. (2015) and Appendix S3 in Supporting Information provide detailed descriptions of the algorithm and methods, respectively, for creating and evaluating ecological regions. Analyses were conducted in MatLab (Release 2015a, The MathWorks, Inc., Natick, MA, USA) and then replicated within the R computing environment (R Core Team 2015) for more accessible use by ecologists. Our R code is publicly available on GitHub for download and can be modified for individual research needs (https://github.com/cont-limno/SpectralClustering4Regions).

**Figure 2:**
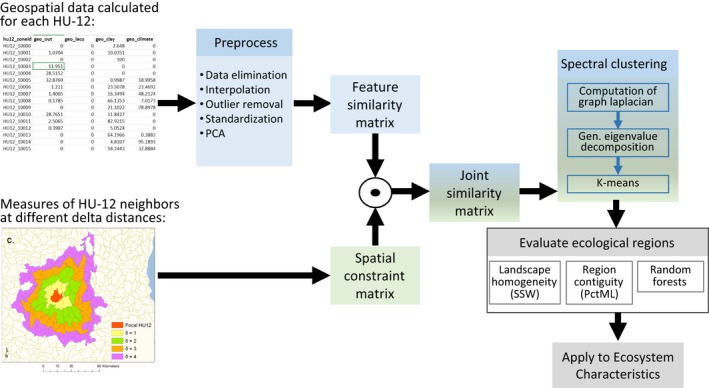
Schematic illustrating the procedure for creating ecological regions from geospatial data using a spatially constrained spectral clustering method, evaluating the ecological regions, and applying them to ecosystem properties. The circle represents the Hadamard product. See text, Appendix S3 and Yuan et al., 2015 for details

Briefly, data were preprocessed to remove HU‐12s that included spatially isolated landscape features (e.g., islands and peninsulas), to fill in missing values in the geospatial database through interpolation, and to remove egregious outliers, all of which could degrade the effectiveness of the clustering algorithm (Figure 2). This preprocessing resulted in 18,856 HU‐12s in the study extent. To reduce bias introduced by geospatial variables with wide ranges and to help account for multicollinearity, geospatial data were standardized and reduced to a smaller number of unweighted, independent variables using Principal Components Analysis (PCA; *n* = 24 axes accounting for 85% of variation; Appendix S3).

Using the PCA scores, we applied the Yuan et al. (2015) method to aggregate HU‐12s into coarser ecological regions. Between every pair of HU‐12s, a landscape feature similarity matrix that measures landscape homogeneity was computed using the Gaussian radial basis function (Buhmann, 2003), and a binary‐valued spatial constraint matrix was constructed based on HU‐12 contiguity (i.e., 1 if the HU‐12s share a border; 0 if the HU‐12s do not share a border; Figure 2). The spatial constraint matrix is used to guide the clustering process into finding spatially contiguous regions. Specifically, the algorithm allows users to specify a parameter, δ, that controls the neighborhood size in which a pair of HU‐12s are required to be in the same region (i.e., as δ increases, the spatial constraint becomes more relaxed; Figure 1; Yuan et al. 2105). These two matrices are merged into a combined similarity matrix through a Hadamard product that includes both landscape homogeneity and spatial contiguity information. The constrained spectral clustering algorithm applied the following two steps to group the HU‐12s into regions: (1) eigenvectors are extracted from the combined similarity matrix using generalized eigenvalue decomposition (von Luxburg, 2007) and (2) *k*‐means clustering is applied to the extracted eigenvectors to obtain the final clusters/regions (Appendix S3).

We evaluated these ecological regions by quantifying the quality of the clustering solution in two ways. First, we quantified the sum‐of‐square error within and between clusters (SSW and SSB, respectively), which represents landscape heterogeneity within and between regions. Regions with low SSW and high SSB have high within‐region homogeneity and high among‐region heterogeneity of geospatial features. The clustering step was repeated 1,000 times because *k*‐means clustering results are sensitive to initialization of cluster centers (Tan, Steinbach, & Kumar, 2005), and we subsequently selected the solution with lowest SSW. Second, we quantified a spatial contiguity metric called “percent of must link” (PctML) that measures the percentage of spatial constraints preserved by the clustering algorithm. A higher value indicates that the resulting regions were more spatially connected (Appendix S3).

#### 2.2.2 Number of regions

A standard approach to choose the optimal number of clusters, *k*, is to plot values of an internal cluster validity index such as SSW against the number of regions and identify the inflection point in the monotonically decreasing curve (e.g., Jain & Dubes, 1988). Unfortunately, this approach is subjective and the inflection point may not always be easily identified. Furthermore, it does not consider the statistical significance of the regions compared to purely random clustering (i.e., no consideration of landscape homogeneity or region contiguity). Worse still, the monotonically decreasing relationship between SSW and number of regions is observed even for purely random clustering (i.e., no consideration of landscape homogeneity or region contiguity).

Therefore, we compared the SSW of the regions created with spatially constrained spectral clustering (SSC; Yuan et al., 2015) against the average SSW for 200 randomly created sets of regions to ensure that the improvement in SSW as the number of regions increases was statistically significant. To do this, we computed the ratio of slopes for the two approaches as the number of regions increases: $\Delta_{\mathrm{slope}}\left(k\right)=\frac{{\text{Slope}}_{\mathrm{SSC}}\left(k-1\right)\;-\;{\text{Slope}}_{\mathrm{SSC}}\left(k\right)}{{\text{Slope}}_{\mathrm{random}}\left(k-1\right)\;-\;{\text{Slope}}_{\mathrm{random}}\left(k\right)}$, where the numerator measures the slope of the SSW curve for the spatially constrained clustering approach and the denominator measures the corresponding slope for the average SSW of the random clustering approach. If the constrained spectral clustering approach provides little improvement in SSW compared to the random clustering approach, then this ratio approaches 1 on plots of empirical curves and indicates an optimal number of regions. We calculated the ratio of the change in slope as the number of regions increased from 5 to 1,000 (with a step size of 5 from 5 to 600 clusters, a step size of 10 from 610 to 800 clusters, and a step size of 50 from 850 to 1,000 clusters) for the spatially constrained spectral clustering approach to that of the “random” clustering approach. Because the empirically estimated ratio of slopes is not always stable, potentially fluctuating around 1 when varying the number of regions near its optimal value, we obtained a more robust estimate by considering an interval of number of regions from *k*‐w/2 to *k*+w/2, where w + 1 is the window size and calculated the average ratio of the change in slope for each window. We then chose the optimal number of clusters to be a value within the first window in which the average ratio of the change in slope is closest to (but not equal to) 1.

#### 2.2.3 Landscape homogeneity and region contiguity

We explored the roles of landscape homogeneity and region contiguity when creating ecological regions and how to balance these two desirable ecological region characteristics. We created nine sets of ecological regions: (1) those made with SSC (Yuan et al., 2015) that account for landscape homogeneity and region contiguity (δ = 4, 4, 8, 16); (2) those made with spectral clustering that ignores landscape homogeneity but uses the same four levels of contiguity; and (3) those made with *k*‐means clustering that ignores contiguity (i.e., no δ), uses solely landscape homogeneity, and is applied directly to the PCA features (as opposed to SSC that applies *k*‐means clustering on the eigenvectors extracted from the combined similarity matrix). We compared these nine sets of ecological regions using the two metrics described above (Yuan et al., 2015): SSW that quantifies the landscape homogeneity within the regions (lower SSW implies higher within‐region homogeneity of geospatial features) and PctML that measures the percentage of spatial constraints preserved by the clustering algorithm (a higher value implies that regions are more spatially contiguous). Thus, ecological regions that are both homogenous and contiguous will have low SSW and high PctML.

### 2.3 Determining terrestrial, climatic, and freshwater drivers of ecological regions

For each of the nine sets of ecological regions, we evaluated the relative importance of the 52 geospatial variables for region formation using a random forest algorithm (Cutler, Edwards, Beard, Cutler, & Hess, 2007) in the R package randomForest (Liaw & Wiener, 2002). Random forest uses random subsets of the data to generate 500 classification trees, and an out‐of‐bag (OOB) error estimate for each tree based on the prediction error for the withheld portion of the dataset. The OOB error estimate was used to identify which geospatial variables were most important for cluster delineation. Importance of each variable was determined by comparing the mean decrease in the Gini impurity criterion, which is a measure of node impurity during classification. Summing the decrease in Gini from parent node to descendent node for each tree indicates variable importance, with large decreases in Gini corresponding to high variable importance (Breiman, Friedman, Stone, & Olshen, 1984; Cutler et al., 2007).

### 2.4 Testing the ability of ecological regions to capture regional variation

We assigned region membership to the approximately 6,000 lakes in LAGOS‐NE_LIMNO_ v1.054.1 (Soranno et al., 2015) for which we had lake total phosphorus and water clarity data. We examined the performance of the nine sets of clusters for capturing broad‐scale variation in ecosystem characteristics not included in creating the ecological regions by examining SSW for the two lake characteristics (lower SSW implies higher within‐region homogeneity of lake characteristics). We also examined the ratio of SSW:SSB in order to compare relative amounts of within‐ and among‐region heterogeneity across response variables and ecological regions. Lower SSW:SSB implies that relatively more heterogeneity in geospatial features, lake total phosphorus, or lake water clarity is among regions than within them.

## 3 Results

### 3.1 Creating ecological regions

#### 3.1.1 Number of regions

**Figure 3:**
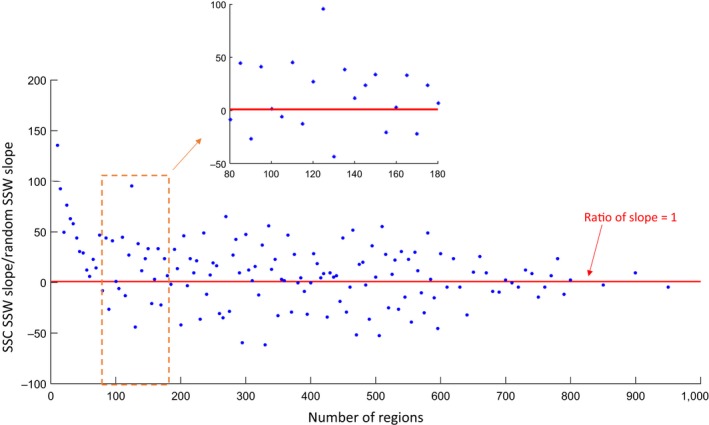
The range of optimal number of regions in our study extent, calculated using the ratio of slopes for the regions created using spatially constrained spectral clustering (SSC) to the slope for the regions created using a completely random clustering approach (no consideration of geospatial features or region contiguity). The inset is a blow‐up of the range of number of regions that included the optimal number (between 80 and 110) for our study extent and geospatial data

We attempted to statistically determine the optimal number of regions to create in our study extent. However, given the large number of HU‐12s to cluster, the resulting SSW was quite high (~10^5^). Thus, even a small change in the slope of SSW for constrained spectral clustering or random clustering could be amplified when calculating the ratio of slopes. We observed significant variability in the ratio of the slopes when the number of regions was greater than 80 (Figure 3). Therefore, we considered an interval of number of regions from *k*‐10 to *k* + 10 with window size 21 and calculated the average ratio of the change in slope for each window. The results indicated the optimal range of region number was between 80 and 110 for our study extent and the geospatial data we included. Based on this result, we created nine sets of 100 ecological regions that averaged 15,842 km^2^ in area for further analysis.

#### 3.1.2 Landscape homogeneity and region contiguity

**Figure 4:**
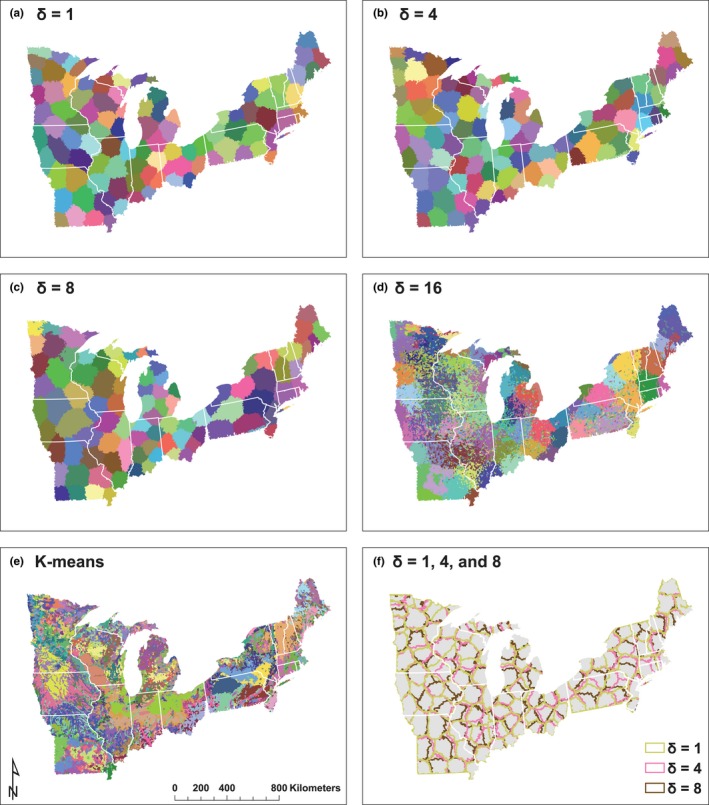
(a–f) Maps depicting the ecological regions created using spatially constrained spectral clustering and varying the level of region contiguity (i.e., the neighborhood constraint δ = 1, 4, 8 and 16; a‐d), as well as *k*‐means clustering on the PCA factors with no contiguity constraint (e) and a combination plot contrasting the boundaries of the δ = 1, 4, and 8 regions (f). White lines indicate U.S. state borders

We mapped region boundaries and HU‐12 region membership across the nine sets of multithemed ecological regions to examine the relative influence of landscape homogeneity and region contiguity in delineating regions (Figure 4). We found relatively similar boundaries and region‐membership when using a strict (δ = 1) to moderate (δ = 4, 8) level of contiguity (Figure 4). In contrast to the very contiguous regions at these strict to moderate levels of contiguity, when region contiguity was weak (δ = 16) the resulting regions appeared more similar to the patchwork of regions, with individual regions distributed across several states, generated by *k*‐means clustering of the PCA features (no contiguity constraint; Figure 4). When regions were created with SSC, which considers both landscape homogeneity and region contiguity, the PctML metric that evaluated contiguity ranged from 12% (at δ = 16) to 90% (at δ = 1) (Table 2A). These values were intermediate between those for regions created with only the contiguity constraint (e.g., constructed with no consideration of landscape homogeneity) and those created with *k*‐means, with no consideration of contiguity (Table 2A).

**Table 2:** Metrics of clustering to create ecological regions. (A) Sum of squares error within regions (SSW), the spatial contiguity metric (PctML, measured in %), and the ratio of the sum of squares within and between regions (SSW:SSB) for ecological regions created using 52 geospatial variables characterizing the terrestrial, climatic, and freshwater landscapes. (B) Sum of squares error within regions **(**SSW) and the ratio of the sum of squares within and between regions (SSW:SSB) for two lake characteristics (mean values). Regions were made with (1) spatially constrained spectral clustering (SSC; Yuan et al., 2015) along a continuum of contiguity created by varying the contiguity constraint (δ = 1, 4, 8, 16), (2) spectral clustering (SC) along that same contiguity continuum while ignoring landscape homogeneity, and (3) *k*‐means clustering (K) of the PCA features directly with no contiguity constraint. “Considers” refer to whether regions were made while accounting for landscape homogeneity, region contiguity, or both. Bolded values point out the ecological regions created with SSC that resulted in the smallest SSW, largest PctML, or smallest ratio. Total sum of squares variation (SSW + SBB) for each response variables was: 836,439, 3,852, and 5,887 for geospatial variables, total phosphorus, and water clarity, respectively. Mod = moderate

(A) Geospatial features								
Clustering method	Considers: Region contiguity	Considers: Landscape homogeneity	Metric	Contiguity level				
				None	Loose	Moderate	Moderate	Strict
				No δ	δ = 16	δ = 8	δ = 4	δ = 1
(1) SSC	Yes	Yes	SSW(PctML)		**379,370** (12)	380,608 (31)	412,407 (60)	447,160 **(90)**
(2) SC	Yes	No	SSW(PctML)		639,402 (57)	559,241 (92)	499,723 (92)	**468,813 (93)**
(3) K	No	Yes	SSW(PctML)	152,220^a^ (45)				
			SSW:SSB	0.22^a^	**0.83**	**0.83**	0.97	1.15

(B) Lake characteristics								
Clustering Method	Considers: Region contiguity	Considers: Landscape homogeneity	Lake characteristic & Metric	Contiguity level				
				None	Loose	Mod	Mod	Strict
				No δ	δ = 16	δ = 8	δ = 4	δ = 1
			Total Phosphorus					
(1) SSC	Yes	Yes	SSW		2,785	**2,591**	2,686	2,619
(2) SC	Yes	No	SSW		3,183	2,815	2,756	**2,478**
(3) K	No	Yes	SSW	2,687^a^				
			SSW:SSB	2.31^a^	2.61	**2.06**	2.30	2.12
			Water Clarity					
(1) SSC	Yes	Yes	SSW		3,935	3,833	**3,831**	3,851
(2) SC	Yes	No	SSW		4,647	4,136	3,924	**3,910**
(3) K	No	Yes	SSW	4,271^a^				
			SSW:SSB	2.64^a^	2.02	1.87	**1.86**	1.89

a This method does not include the region contiguity constraint.

The goal when creating regions is to create contiguous regions that are homogeneous (i.e., that minimize the heterogeneity within regions; SSW). At one end of the spectrum, clustering while ignoring contiguity created regions with the lowest SSW (Table 2A), thus confirming that the best way to create clusters of homogeneous geospatial features is to not impose a contiguity constraint. At the other end of the spectrum, clustering with the strictest level of contiguity (*δ* = 1) created regions that were completely contiguous but had high SSW, whether or not a landscape homogeneity constraint was included (Table 2A). In the middle of the contiguity spectrum (δ = 16, 8, 4), regions that considered landscape homogeneity had lower within‐region heterogeneity (SSW) than did those created using only the contiguity constraint.

When considering the cluster performance metrics across the ecological regions created using SSC, the relative amount of variation within regions decreased as contiguity decreased (SSW:SSB decreased as δ increased; Table 2A). The variation within regions was higher than among regions at δ = 1 and a δ = 16 resulted in very noncontiguous regions (PctML = 12% Table 2A; Figure 4d) that are likely not useful for many science and management applications. Therefore, a moderate level of contiguity (δ = 4, 8) appears to best accommodate these two desirable traits of ecological regions for this study extent and number of regions, resulting in, respectively, 95 and 88 of the 100 regions being completely contiguous. Shapefiles of the ecological regions created with δ = 4 and 8 and the R code to reproduce these ecological regions or to modify them for other uses is available in a data repository and on GitHub (Cheruvelil et al., 2016; https://github.com/cont-limno/SpectralClustering4Regions), respectively.

### 3.2 Determining terrestrial, climatic, and freshwater drivers of ecological regions

**Figure 5:**
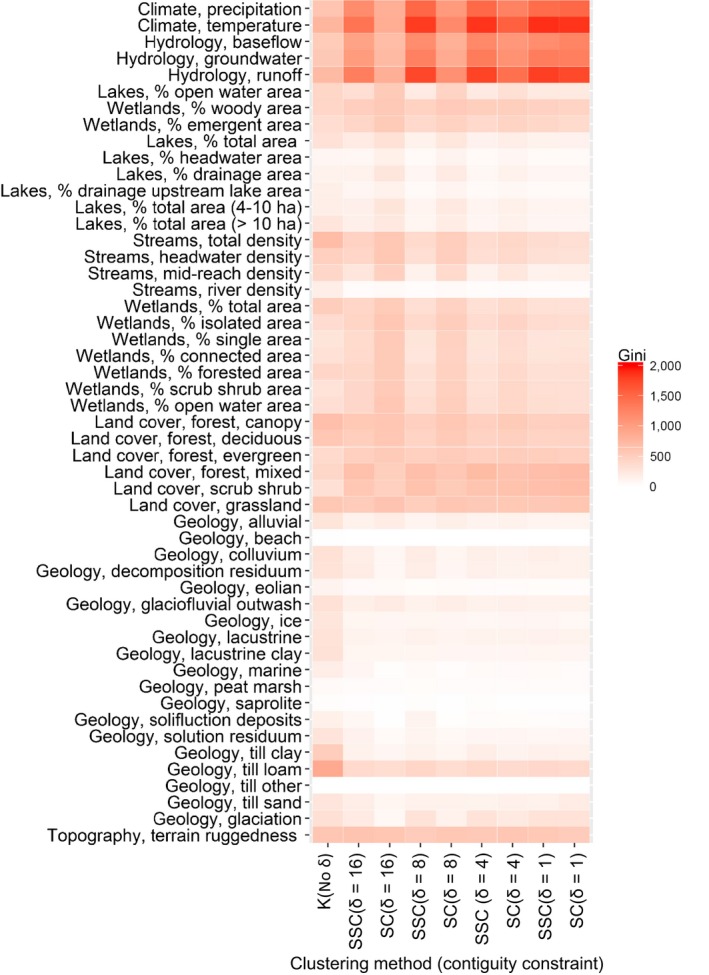
Random forest importance scores heat map for each of the 52 geospatial features and the nine sets of ecological regions created. Values are mean decreases in the Gini impurity criterion, with higher values (darker shading) indicating higher variable importance in the random forest. Regions were made with (1) *k*‐means clustering (K) of the PCA features directly with no contiguity constraint, (2) spatially constrained spectral clustering (SSC; Yuan et al., 2015) along a continuum of contiguity created by varying the contiguity constraint (δ = 1, 4, 8, 16), and (3) with spectral clustering (SC) along that same contiguity continuum while ignoring landscape homogeneity

The geospatial features associated with the ecological regions were largely consistent across the nine sets of 100 ecological regions, as determined by random forest analysis. Geospatial variables from all three themes (i.e., terrestrial, climatic, and freshwater) were important in creating regions for all nine sets of 100 ecological regions across our study extent (Figure 5, Appendix S3). Those variables associated with broad‐scale spatial patterns and gradients, such as precipitation and hydrology, were most strongly associated with regions that had stricter contiguity constraints (δ = 1, 4, 8; dark shading in Figure 5), whereas variables with fine‐scale spatial patterns, such as geology, were most strongly associated with regions created using weak or absent contiguity constraints (δ = 16 or *k*‐means, respectively; little to no shading in Figure 5). Note that when contiguity was strictest (δ = 1), random forest results were extremely similar regardless of whether landscape features were included (SSC) or not (random) because there are a limited number of ways to cluster the HU‐12s when contiguity is strict. Measures of the freshwater and terrestrial landscapes, such as wetland and stream density; land cover; surficial geology of type till loam; and terrain ruggedness were also frequently important for creating regions (Figure 5, Appendix S3). The fact that a combination of terrestrial, climatic, and freshwater landscape features created these ecological regions demonstrates the importance of considering a wide suite of geospatial variables in such efforts.

### 3.3 Testing the ability of ecological regions to capture regional variation

We examined how much of the total variation in lake phosphorus and lake water clarity was attributable to the regional scale and how that changed depending upon whether the clustering method considered landscape homogeneity and the strictness of region contiguity (δ = 1, 4, 8, 16). Results were similar for the two lake characteristics (Table 2B). Interestingly, generating multithemed ecological regions that minimize SSW for geospatial variables (*k*‐means clusters) did not translate to minimizing SSW or SSW:SSB for lake phosphorus and water clarity in the northeastern U.S. In fact, the lowest SSW:SSB was for the ecological regions created with constrained spectral clustering and moderate levels of contiguity (δ = 4 or 8; Table 2B). These ecological regions captured approximately 40% of the variation in these two ecosystem characteristics. These results demonstrate the importance of testing assumptions that underlie region delineation before using them to capture macroscale spatial variation in ecosystem properties and relationships.

## 4 Discussion

This paper describes a test of a novel approach to create ecological regions that balance geospatial homogeneity and region contiguity, two important characteristics of ecological regions. We applied this objective method to a wide range of terrestrial, climatic, and freshwater characteristics that comprise the geographically diverse and expansive landscape setting influencing ecosystems and tested its ability to capture regional variation in lake characteristics. We found that a combination of terrestrial, climatic, and freshwater geospatial features influenced region creation. This result suggests that the oft‐ignored freshwater landscape provides novel information on landscape variability that is not captured by traditionally used climate and terrestrial metrics and reinforces the importance of considering multiple landscape themes and a wide suite of geospatial variables when creating ecological regions. We also found that the delineated regions captured macroscale heterogeneity in two important ecosystem characteristics that were not included in region delineation. Approximately 40% of the total among‐lake variation in total phosphorus and water clarity was captured by the regional scale. Further, these regions captured more regional heterogeneity than did regions created without a contiguity constraint. Our results have several implications for the development and integration of robust ecological regions for effective macrosystems research.

### 4.1 Creating ecological regions

When creating ecological regions in the past, it proved challenging to objectively determine the number of regions to create for a study extent. Statistics and computer science approaches provide ways to do so (Jain & Dubes, 1988; Rousseuw, 1987; Sugar & James, 2003; Tibshirani, Walther, & Hastie, 2001), but they are mostly designed for unconstrained clustering algorithms. In this work, we present a new method that included information about the relative performance of spatially constrained spectral clustering as compared to completely random clustering (i.e., not considering landscape homogeneity or region contiguity). Our empirical results identified a range of optimal regions, falling between 80 and 110. These results highlight that, although regions themselves were generated using objective, data‐driven algorithms, there may be no completely objective way to determine the number of regions. Therefore, the open source code that we provide has the flexibility to create any number of regions to best meet future users’ research or management needs.

Although region contiguity is desirable, most objective clustering algorithms optimize landscape homogeneity over contiguity (Duque et al., 2007; Olden et al., 2012). Creating ecological regions in three ways allowed us to contrast regions that considered landscape homogeneity only (no contiguity constraint; *k*‐means clustering of PCA factors), those that included a constraint for region contiguity only (no consideration of landscape homogeneity; spectral clustering), and those that considered both desirable region features (spatially constrained spectral clustering, SSC; Yuan et al., 2015). Our results indicated a large trade‐off between maximizing either landscape homogeneity using *k*‐means clustering or maximizing region contiguity using a strict level of contiguity (δ = 1) and no landscape homogeneity constraint. However, we found that moderate levels of region contiguity (δ = 4, 8) balanced these two important characteristics of ecological regions and resulted in very few noncontiguous regions (5 and 12, respectively). Therefore, we suggest that a moderate level of region contiguity may be most useful for applications that desire region contiguity while maintaining within‐region homogeneity. Future users can test additional contiguity constraints (e.g., δ = 5, 6, 7) and specify the level of contiguity that best fits their needs.

### 4.2 Determining terrestrial, climatic, and freshwater drivers of ecological regions

Most existing ecological regions were based on the few terrestrial and climatic characteristics that were quantified and available across continental or global extents. Increased data availability allowed us to create regions using data derived from many high‐resolution, continental‐scale datasets that spanned terrestrial, atmospheric, and freshwater themes. We found that all three themes played a role in creating ecological regions across our study extent. Even measures of the oft‐ignored freshwater landscape, such as wetland and stream density (Soranno et al., 2015), played a role in creating regions. Thus, considering measures of the freshwater landscape when creating ecological regions may be important for macroscale studies, especially those focused on freshwaters or biota that rely on freshwaters. Geospatial variables also have different spatial structures (Fortin & Dale, 2005); therefore, we might expect different geospatial variables to drive region creation depending on the size of the spatial extent. For example, we found that two variables that operate on broad‐scales, precipitation and hydrology, were important for creating regions for our spatial extent. These two variables may not be as important for a smaller geographic area, such as an individual state.

We created ecological regions with only natural geospatial variables. Many of the variables that created our ecological regions have been included in past regionalization efforts (e.g., land cover, surficial geology, terrain ruggedness; Cheruvelil et al., 2013). Some existing ecological regions include anthropogenically driven variables (e.g., Omernik, 1987; USDA 2006). However, many questions and applications using ecological regions explicitly examine the effects of anthropogenic activities on ecosystems. Therefore, we did not include anthropogenically driven variables, such as land use, atmospheric deposition, and road density when creating ecological regions. Because anthropogenic drivers strongly affect in‐lake characteristics, we may have found higher among‐region variation in lake nutrients and clarity had we included these drivers when creating ecological regions. However, natural ecological regions can facilitate better understanding of the effects of anthropogenic drivers, each with its own spatial structure, on ecosystem properties and relationships. For example, these drivers can be added to empirical models that include the natural ecological regions to determine how much of the local and regional variation among lakes is explained by individual human disturbances. Finally, many of the natural geospatial variables, such as precipitation and hydrology, are dynamic and highly affected by global climate change and land use intensification. Therefore, we provide freely available and easily accessible code and documentation for this clustering approach to create ecological regions so that users can include anthropogenic variables when custom‐making their ecological regions if it meets their research goals, as well as to remake regions with new data as it becomes available.

### 4.3 Testing the ability of ecological regions to capture regional variation

When including ecological regions in their work, users do so under the assumption that the response variable or relationship of interest will be more similar within regions than among regions, just as the geospatial variables used to create the regions were. Our results generally support this idea. Ecological regions with a moderate level of contiguity better‐grouped similar northeastern and midwest U.S. lakes than did regions created by clustering without a contiguity constraint, with regions accounting for approximately 40% of the total variation in lake total phosphorus and water clarity. This amount of among‐region variance is intermediate to that found by previous studies using different ecological regions and study extents (Cheruvelil et al., 2008, 2013) and is logical for two reasons. First, there are many fine‐scale features (e.g., lake depth) that are known to be important drivers of within‐lake processes that influence nutrients and clarity. Second, we did not a priori select a subset of geospatial features known to influence lake characteristics for creating regions (i.e., we did not develop a customized regionalization framework based on our question). Future research to better identify the role of different landscape drivers in creating regions that capture macroscale variation could include a wider suite of ecosystem types, response variables, geographic study areas, and study extents. For example, as stream and wetland variables were important for creating these ecological regions, it would be interesting to test whether a larger proportion of variation would be at the among‐region scale for stream or wetland response variables.

## 5 Conclusions

Our ecological regions captured macroscale variation in lake nutrients and water clarity, providing evidence of their usefulness for macroscale science and management. Although new data and methods for creating ecological regions exist, relatively recent attempts at creating such ecological regions have not yet been widely adopted (e.g., Hargrove & Hoffman, 2004; Higgins, Bryer, Khoury, & Fitzhugh, 2005; Keller, Schimel, Hargrove, & Hoffman, 2008). One reason for this fact may be the difficulty in changing broad‐scale monitoring and assessment efforts that have been institutionalized (i.e., setting nutrient criteria in the U.S.; U.S. EPA 1998) and the difficulty that such changes could present for long‐term comparisons. Additionally, some newer methods for creating regions were not developed by ecologists, making novel methods difficult for ecologists and managers to find and apply. For example, methodologies for creating regions can be found published in computer science or geography venues and as part of proprietary software (e.g., Hargrove & Hoffman, 1999; Kupfer & Gao, 2012; Stepinski et al., 2015), and code to reproduce or customize those regions are often nonexistent or difficult to find.

To further progress macrosystems ecology research, we provide ecologists with an objective, reproducible, and flexible method for creating ecological regions that will meet a variety of user needs and are freely available (https://github.com/cont-limno/SpectralClustering4Regions). Importantly, almost any spatial data could be used to create regions this way, and as new geophysical data underlying region delineation become available, the method can be rerun to create new regions. Finally, future users can create different numbers/sizes of regions; use a subset of themes or add an anthropogenic theme; use different combinations of geospatial variables; create regions for a different spatial extent (e.g., state, nation, and continent); use different levels of region contiguity; or use regions for capturing broad‐scale variation among different ecosystems or in response to stressors. Such work should facilitate regional‐continental scale understanding of macroscale patterns and processes and predictions of future responses to global change.

## Conflict of Interest

None declared.

## Data Accessibility

We make our data and metadata freely available through the LTER Data Portal (Cheruvelil et al., 2016). Ecological region shapefiles and the R scripts for the constrained spectral clustering algorithm are freely available via a data repository and GitHub (Cheruvelil et al., 2016; https://github.com/cont-limno/SpectralClustering4Regions), respectively.

## Author Contributions

The paper was conceived of and outlined at a January 2014 project meeting that all authors attended, except SMC and JFL. Many of the analyses were conducted during a February 2015 working group of all authors except CTF. All coauthors contributed refinements to the focus of the paper between the two workshops and after the second workshop, performed critical reviews of the manuscript prior to submission, and reviewed and edited the final manuscript. KSC coordinated the writing and revision of the manuscript and wrote the introduction, results, and discussion. SY and PNT conducted all of the computer science analyses (constrained spectral clustering in MatLab) and wrote those methods. SMC, CES, CEF, and KEW conducted the random forest analyses, wrote those methods, and made the heat map figures. ENH, JFL, and CEF conducted descriptive statistics and made the associated tables. KEW made the maps. The QAQC methods development and analysis on LAGOS‐NE_GIS_ were led by CES, SMC, and CEF. The conceptual foundation for measuring freshwater connectivity was led by CEF. The process of converting the ecological regions code into R and documenting that code was aided by KSC, SY, KEW, PNT, and CTF.
