# Supplementary material for: Creating multithemed ecological regions for macroscale ecology: Testing a flexible, repeatable, and accessible clustering method
Source: Ecol Evol. 2017 Mar 26;7(9):3046–58. doi: 10.1002/ece3.2884 (PMC5415510; doi:10.1002/ece3.2884)
Supplement: Supplementary file 1 [file ECE3-7-3046-s001.docx]

**List of Supporting Information and Appendices:**

**Appendix S1**. Table of geospatial data, including variables names as depicted in figures, units, descriptions and sources, and descriptive statistics.

**Appendix S2.** Map of LAGOS-NE_LIMNO_ v1.054.1 lakes used in the case study indicating (a) lake water clarity (Secchi disk depth, m) and (b) lake total phosphorus (µg L^-1^). Bins were derived from quartile values in Table 1.

**Appendix S3.** Detailed methods for creating and evaluating ecological regions with constrained spectral clustering

**Appendix S1**. Table of geospatial data, including variables names as depicted in figures, units, descriptions and sources, and descriptive statistics.

| **Variable name** | **Theme** | **Units** | **Description** | **Median** | **Mean** | **Max** | **Min** | **25th** | **75th** | **n** |
| --- | --- | --- | --- | --- | --- | --- | --- | --- | --- | --- |
| Climate, precipitation^1^ | CL | mm | Mean of the 30-yr long-term (normal) annual mean precipitation for zone | 980.1 | 975.8 | 1691.6 | 536.2 | 846.6 | 1106.6 | 18832 |
| Climate, temperature^1^ | CL | C | Mean of the 30-yr long-term (normal) annual mean temperature for zone | 8.8 | 8.7 | 15.5 | 2.4 | 6.8 | 10.8 | 17160 |
| Hydrology, baseflow^2^ | FW | % | Mean value of % of streamflow that can be attributed to ground-water discharge into streams in the zone (baseflow/total flow *100) | 45.2 | 43.7 | 89.9 | 6.8 | 33.0 | 54.1 | 16538 |
| Hydrology, groundwater^3^ | FW | mm/yr | Mean of mean annual natural groundwater recharge in zone - baseflow multiplied by mean annual runoff | 114.6 | 149.8 | 506.7 | 7.9 | 72.1 | 223.1 | 18349 |
| Hydrology, runoff^4^ | FW | in/yr | Mean value of average annual runoff in the zone, 1951-80 | 11.6 | 13.2 | 40.3 | 0.9 | 8.5 | 17.7 | 17910 |
| Lakes, % open water area5 | FW | % | Percent of land use/cover in the zone from the 1992 NLCD, Open Water | 0.5 | 2.3 | 99.7 | 0.0 | 0.1 | 1.9 | 12131 |
| Wetlands, % woody area^5^ | FW | % | Percent of land use/cover in the zone from the 2001 NLCD, Woody Wetlands | 1.3 | 6.0 | 95.9 | 0.0 | 0.1 | 7.1 | 14074 |
| Wetlands, % emergent area5 | FW | % | Percent of land use/cover in the zone from the 2001 NLCD, Emergent Herbaceous Wetland | 0.2 | 1.5 | 64.0 | 0.0 | 0.0 | 1.2 | 10164 |
| Lakes, % total area6 | FW | % | The percent of the zone that is "covered" by lakes >=4 ha | 0.1 | 1.6 | 99.7 | 0.0 | 0.0 | 0.8 | 8438 |
| Lakes, % isolated area6 | FW | % | The percent of the zone that is covered by isolated (no inflow or outflow streams) lakes >=4 ha | 0.0 | 0.2 | 27.1 | 0.0 | 0.0 | 0.1 | 3682 |
| Lakes, % headwater area6 | FW | % | The percent of the zone that is covered by headwater (no inflowing streams) lakes >=4 ha | 0.0 | 0.1 | 36.8 | 0.0 | 0.0 | 0.0 | 3125 |
| Lakes, % drainage area6 | FW | % | The percent of the zone that is covered by DRStream lakes >=4 ha | 0.0 | 0.4 | 77.3 | 0.0 | 0.0 | 0.2 | 5517 |
| Lakes, % drainage upstream lake area6 | FW | % | The percent of the zone that is covered by DRLakeStream lakes >=4 ha | 0.0 | 0.9 | 99.7 | 0.0 | 0.0 | 0.0 | 4016 |
| Lakes, % total area (4-10 ha)6 | FW | % | The percent of the zone that is covered by lakes 4 to 10 ha | 0.0 | 0.1 | 8.8 | 0.0 | 0.0 | 0.1 | 3776 |
| Lakes, % total area (> 10 ha)6 | FW | % | The percent of the zone that is "covered" by lakes >=10 ha | 0.0 | 1.5 | 99.7 | 0.0 | 0.0 | 0.7 | 7171 |
| Streams, total density^6^ | FW | m/ha | The sum of all streams (NHDFlowline minus artifical lines through lakes) within the zone divided by the area of the zone | 11.2 | 11.4 | 70.3 | 0.0 | 7.7 | 14.4 | 17782 |
| Streams, headwater density6 | FW | m/ha | The sum of all headwater streams within the zone divided by the area of the zone | 9.8 | 10.0 | 59.2 | 0.0 | 6.6 | 12.8 | 17739 |
| Streams, mid-reach density6 | FW | m/ha | The sum of all midreach streams within the zone divided by the area of the zone | 1.1 | 1.3 | 26.0 | 0.0 | 0.0 | 2.1 | 11289 |
| Streams, river density6 | FW | m/ha | The sum of all large rivers within the zone divided by the area of the zone | 0.0 | 0.1 | 7.9 | 0.0 | 0.0 | 0.0 | 1285 |
| Wetlands, % total area^7^ | FW | % | The percent of the zone that is "all wetlands" | 2.8 | 7.4 | 99.8 | 0.0 | 0.8 | 9.4 | 16843 |
| Wetlands, % isolated area7 | FW | % | The percent of the zone that is "isolated wetlands" | 0.6 | 2.6 | 78.1 | 0.0 | 0.1 | 2.8 | 12727 |
| Wetlands, % single area7 | FW | % | The percent of the zone that is "single wetlands" | 0.2 | 0.9 | 29.2 | 0.0 | 0.0 | 1.1 | 9996 |
| Wetlands, % connected area7 | FW | % | The percent of the zone that is "connected wetlands" | 1.7 | 3.9 | 85.7 | 0.0 | 0.4 | 4.7 | 15421 |
| Wetlands, % forested area7 | FW | % | The percent of the zone that is "forested wetlands" | 1.4 | 4.3 | 79.8 | 0.0 | 0.3 | 4.9 | 15005 |
| Wetlands, % scrub shrub area7 | FW | % | The percent of the zone that is "scrub shrub wetlands" | 0.2 | 1.6 | 81.9 | 0.0 | 0.0 | 1.5 | 10149 |
| Wetlands, % open water area7 | FW | % | The percent of the zone that is "open water wetlands" | 0.4 | 1.5 | 75.0 | 0.0 | 0.1 | 1.4 | 11732 |
| Land cover, forest, canopy5 | TR | % | Mean tree canopy percent (in 2001) for zone | 28.6 | 34.3 | 96.0 | 0.0 | 9.8 | 58.3 | 18594 |
| Land cover, forest, deciduous5 | TR | % | Percent of land use/cover in the zone from the 2001 NLCD, Deciduous Forest | 20.3 | 25.5 | 93.3 | 0.0 | 7.7 | 39.5 | 18490 |
| Land cover, forest, evergreen5 | TR | % | Percent of land use/cover in the zone from the 2001 NLCD, Evergreen Forest | 0.5 | 4.0 | 71.6 | 0.0 | 0.0 | 4.2 | 11725 |
| Land cover, forest, mixed5 | TR | % | Percent of land use/cover in the zone from the 2001 NLCD, Mixed Forest | 0.2 | 4.5 | 60.3 | 0.0 | 0.0 | 3.7 | 10370 |
| Land cover, scrub shrub5 | TR | % | Percent of land use/cover in the zone from the 2001 NLCD, Scrub/Shrub | 0.3 | 1.4 | 46.0 | 0.0 | 0.0 | 1.5 | 10317 |
| Land cover, grassland5 | TR | % | Percent of land use/cover in the zone from the 2001 NLCD, Grassland/Herbaceous | 0.8 | 1.8 | 52.1 | 0.0 | 0.3 | 2.1 | 13541 |
| Geology, alluvial^8^ | TR | % | The percent of the zone that is alluvial | 0.0 | 4.7 | 100.0 | 0.0 | 0.0 | 0.0 | 4570 |
| Geology, beach8 | TR | % | The percent of beach deposits of Holocene age | 0.0 | 0.0 | 26.9 | 0.0 | 0.0 | 0.0 | 57 |
| Geology, colluvium8 | TR | % | The percent of all colluvium of all ages | 0.0 | 7.8 | 100.0 | 0.0 | 0.0 | 0.0 | 1683 |
| Geology, decomposition residuum8 | TR | % | The percent of decomposittion residuum of all ages | 0.0 | 4.1 | 100.0 | 0.0 | 0.0 | 0.0 | 1481 |
| Geology, eolian8 | TR | % | The percent of eolian silt (loess), sand of all ages | 0.0 | 1.1 | 100.0 | 0.0 | 0.0 | 0.0 | 929 |
| Geology, glaciofluvial outwash8 | TR | % | The percent of glaciofluvial (outwash) deposits of all ages | 0.0 | 8.2 | 100.0 | 0.0 | 0.0 | 1.9 | 5297 |
| Geology, ice8 | TR | % | The percent of ice-contact depositives of late Wisconsin and Holocene ages | 0.0 | 3.8 | 100.0 | 0.0 | 0.0 | 0.0 | 2502 |
| Geology, lacustrine8 | TR | % | The percent of lacustrine (combination of the following categories: glacial-lake clay and silt lake deposits of pre-Illinoian and Illinoin age; glacial and postglacial lake deposits) | 0.0 | 4.0 | 100.0 | 0.0 | 0.0 | 0.0 | 2104 |
| Geology, lacustrine clay8 | TR | % | The percent of lacustrine clay (glacial and postglacial lake deposits (clay and silt) of Holocene and late Wisconsin age) | 0.0 | 1.4 | 100.0 | 0.0 | 0.0 | 0.0 | 400 |
| Geology, marine8 | TR | % | The percent of coastal-plain marine deposits of Pleistocene and Pliocene ages, and glaciomarine deposits of late Wisconsin age | 0.0 | 1.2 | 99.1 | 0.0 | 0.0 | 0.0 | 1018 |
| Geology, peat marsh8 | TR | % | The percent of peat marsh (coastal or inland deposits of freshwater, brackish-water, peat and muck or freshwater swamps of Holocene and late Wisconsin age) | 0.0 | 1.3 | 100.0 | 0.0 | 0.0 | 0.0 | 161 |
| Geology, saprolite8 | TR | % | The percent of saprolite of quaternary and tertiary age on crystalline iigneous and metamorphic rocks mostly | 0.0 | 3.2 | 100.0 | 0.0 | 0.0 | 0.0 | 1173 |
| Geology, solifluction deposits8 | TR | % | The percent of solifluction deposits of Holocene and late to middle Pleistocene age, and lake Wisconsin age | 0.0 | 7.0 | 100.0 | 0.0 | 0.0 | 0.0 | 2003 |
| Geology, solution residuum8 | TR | % | The percent of solution residuum of Quaternary and tertiary age | 32.4 | 41.3 | 100.0 | 0.0 | 0.0 | 84.9 | 9270 |
| Geology, till clay8 | TR | % | The percent of till, clay of pre-Illinoian age, late Wisconsin and Holocene ages | 0.0 | 0.0 | 100.0 | 0.0 | 0.0 | 0.0 | 17 |
| Geology, till loam8 | TR | % | The percent of till, loam of pre-Illinoian, Illinoian, and late Wisconsin and Holocene ages | 0.0 | 7.7 | 100.0 | 0.0 | 0.0 | 0.0 | 1663 |
| Geology, till other8 | TR | % | The percent of till, complex depositions of lake Wisconsin and Illinoian age | 1.0 | 1.8 | 3.0 | 1.0 | 1.0 | 3.0 | 3 |
| Geology, till sand8 | TR | % | The percent of till, sand of pre-Illinoian age, late Wisconsin and Holocene ages | 0.5 | 2.3 | 99.7 | 0.0 | 0.1 | 1.9 | 12131 |
| Geology, glaciation8 | TR | n/a | Categorical value of late Wisconsonian glaciation; Glaciated=entire zone glaciated, Partially_Glaciated=zone has a mix of glaciated and not-glaciated land, Not_Glaciated=entire zone not glaciated | 1.3 | 6.0 | 95.9 | 0.0 | 0.1 | 7.1 | 14074 |
| Topography, terrain ruggedness^9^ | TR | m | The terrain ruggedness index (TRI) at each cell is the TRI with respect to its immediate neighbors (10m scale). The mean is the mean cell value for the TRI variable for each zone. | 0.4 | 0.5 | 2.9 | 0.0 | 0.2 | 0.7 | 9386 |

^1^ PRISM climate group, http://www.prism.oregonstate.edu

^2^ USGS, https://catalog.data.gov/dataset/base-flow-index-grid-for-the-conterminous-united-states

^3^ USGS, https://catalog.data.gov/dataset/estimated-mean-annual-natural-ground-water-recharge-in-the-conterminous-united-states

^4^ USGS, https://catalog.data.gov/dataset/average-annual-runoff-in-the-united-states-1951-80

^5^ NLCD, http://www.mrlc.gov

^6^ USGS, http://nhd.usgs.gov

^7^ USFWS, http://www.fws.gov/wetlands/data

^8^ USGS, http://pubs.usgs.gov/imap/i-2789/

^9^ USGS, http://ned.usgs.gov/

**Appendix S2.** Map of LAGOS_LIMNO_ v1.054.1 lakes used in the case study indicating (a) lake water clarity (Secchi disk depth, m) and (b) lake total phosphorus (µg L^-1^). Bins were derived from quartile values in Table 1.


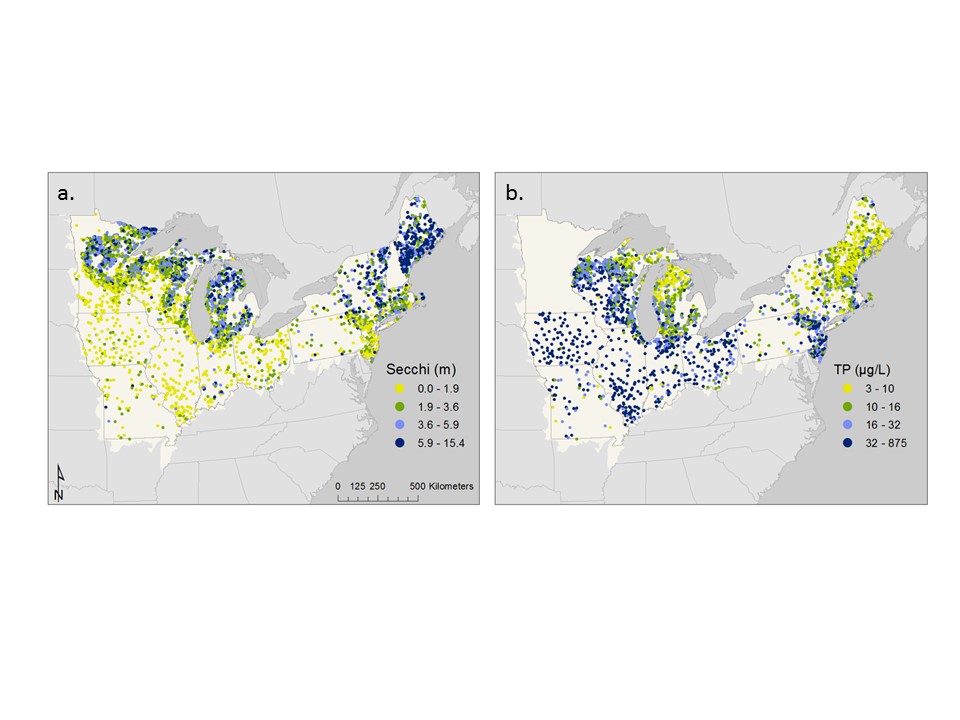


**Appendix S3.** Detailed methods for creating and evaluating ecological regions with constrained spectral clustering

Ecological regions were created using a constrained spectral clustering method that considers both landscape homogeneity and region contiguity (spatially constrained spectral clustering, SSC; Yuan et al. 2015). Figure 2 provides a schematic illustration of the method.

The input data was initially preprocessed to remove outlying HU-12s, such as those that contained unique geographic features or were spatially disconnected from other HU-12s (e.g., islands and peninsulas). After preprocessing, we omitted 1,378 HU-12s that bordered our study extent and were predominantly contained in Canada or bordering U.S. states, three HU-12s that were islands in Lake Ontario and the St. Lawrence Seaway, one HU-12 that acted as an island at the edge of the Pennsylvanian (i.e., surrounded on three sides by a river and on the forth side by the study extent border), and 19 HU-12s on two peninsulas that were connected to the mainland by bridges (i.e., Cape Cod, Massachusetts, and Door County Peninsula, Wisconsin). These island and peninsula HU-12s were subsequently assigned to the same region as their nearest mainland HU-12 after the clustering had completed. The resulting dataset contained 18,856 HU-12s.

The list of geospatial variable names (n=52), themes, sources, and descriptive statistics calculated are given in Appendix S1. Seventy-nine HU-12s required additional preprocessing in order to impute their missing values, eliminate outliers, and address other data-related issues that could degrade the effectiveness of the clustering algorithm. First, there were 70 missing values that were imputed by interpolating with the average value of immediately neighboring HU-12s. Next, we eliminated nine outlier HU-12s using the 1-nearest neighbor approach (Knorr et al. 2000). Specifically, if the feature similarity of a HU-12 to its most similar HU-12 was low (i.e., below a minimum similarity threshold of 0.2), then the HU-12 was considered an outlier. Similar to the island HU-12s, these outlier HU-12s were re-assigned to the same region as its geographically nearest HU-12 after the clustering had completed. These preprocessing steps affected 0.004% of the HU-12 data.

Next, we standardized the variables by subtracting the mean value and dividing by the standard deviation. To account for multicollinearity among features, we applied principal component analysis and extracted the smallest number of independent components that collectively explained a total of at least 85% of the variation in the data. These 24 PCA components (Figure S2-1) were then used for clustering.


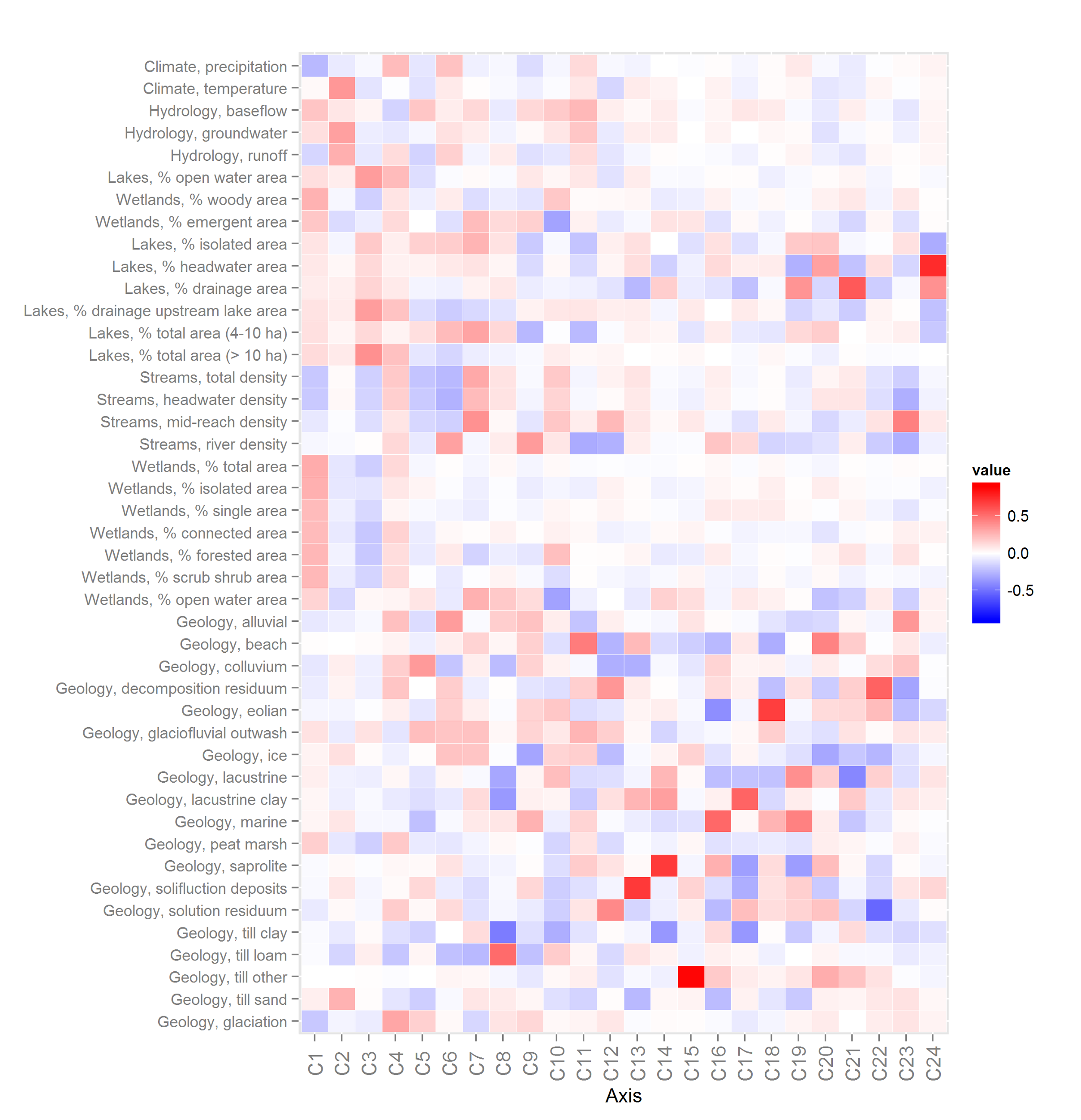


**Figure S3-1.** Heat map depicting the results of the PCA conducted as part of the preprocessing before creating the regions with constrained spectral clustering. Value refers to loadings on PC axes.

We aggregated the HU-12s into coarser, spatially contiguous regions by applying the spatially constrained spectral clustering algorithm described in (Yuan et al. 2015) to the PCA scores. The algorithm was designed to produce spatially contiguous clusters while maximizing the landscape homogeneity within the regions. This was accomplished by allowing users to specify pairs of spatially adjacent HU-12s that should be assigned to the same region. These pairwise constraints were used to guide the clustering process into finding regions that are homogeneous and spatially contiguous.

A landscape feature similarity matrix is computed between every pair of HU-12s using the Gaussian radial basis function (Buhmann 2003). The similarity function is calculated as follows: $S_{ij}^{feature}=\exp\left( -\frac{\left\| x_{i}-x_{j} \right\|^{2}}{2\sigma^{2}} \right)$, where $x_{i}$ and $x_{j}$ are the feature vectors associated with the *i^th^* and *j^th^* HU-12s while σ is the kernel width parameter, set to be the average Euclidean distance of the feature vectors for all pairs of HU-12s. A spatial constraint matrix C is also constructed, where $C_{ij}=1$ if the *i^th^* and *j^th^* HU-12s share a boundary; otherwise $C_{ij}=0$. Instead of using the constraint matrix directly, the algorithm transforms the constraint matrix into a binarized truncated exponential kernel matrix, $S^{const}= I\left[ \sum_{i=0}^{\delta} C^{i}>0 \right]$, where $\delta$ is a parameter that controls the neighborhood size in which a pair of HU-12s are required to be in the same region (Yuan et al. 2015). If $\delta=1$, then only the immediate neighbors (i.e., spatially adjacent HU-12s) are constrained to be in the same region (Figure 1c). If $\delta=2$, then the spatial constraint also includes neighbors that are adjacent to the immediate neighbors of a HU-12. As$\delta$ increases, the spatial constraint becomes more relaxed, allowing HU-12s that are located far away from a given HU-12 to be in the same region (see Figure 1c for a visual representation).

As shown in Figure 2, the constrained spectral clustering algorithm combines the feature similarity matrix ($S_{ij}^{feature}$) with the binarized truncated exponential kernel matrix ($S_{ij}^{const}$) to form a joint similarity matrix, $S_{ij}^{total}= S_{ij}^{feature} \circ S_{ij}^{const}$. This element-wise matrix product, also known as a Hadamard product, creates a sparse joint matrix by setting the similarity between pairs of HU-12s located far away from each other to zero. This prevents HU-12s located far away from each other from being assigned to the same region without artificially inflating the similarity between pairs of HU-12s that are close to each other (Yuan et al. 2015).

We applied a spectral clustering algorithm to the joint similarity matrix. The solution to the spectral clustering algorithm can be found by solving a generalized eigenvalue problem, $L^{total}r= \lambda D^{total}r$, where $L^{total}=D^{total}-S^{total}$ and is the graph Laplacian associated with the joint similarity matrix, and $D^{total}$is a diagonal matrix whose non-zero diagonal elements are equal to the row sums in $S^{total}$ (Luxburg 2007). The eigenvectors that correspond to the top K smallest eigenvalues are computed and provided as input features to the k-means clustering algorithm to generate the final cluster membership. The value of k depends on the number of clusters to be extracted (see below for the method for choosing k). Since these k-means clustering results are sensitive to initialization of their cluster centers (Tan et al. 2005), we repeated the clustering step 1000 times and chose the solution with lowest within-cluster sum-of-square errors. We compared the regions created using constrained spectral clustering (with $\delta=1, 4, 8,16)$against those created using the spatial contiguity matrix only as well as those based solely on landscape homogeneity (k-means clusters).

We considered two metrics for evaluating a regionalization framework based on the quality of the clustering solution (Yuan et al. 2015). First, we computed the within-cluster sum-of-square error (SSW) to quantify the landscape homogeneity within the regions, $SSW= \sum_{i=1}^{k} \sum_{x \in C_{i}} dist\left( \mu_{i}, x \right)^{2}$, where $\mu_{i}$ is the centroid of cluster $C_{i}.$A lower SSW implies higher homogeneity of landscape features within regions. The second metric corresponds to a measure of cluster contiguity, which is computed based on the percentage of spatial constraints preserved by the clustering algorithm, $PctML= \frac{\# ML edges within discovered regions}{Total \# of ML edges}$. The numerator corresponds to the number of must links (i.e. spatial constraints) preserved within each region while the denominator corresponds to the total number of must links (non-zeros) in the spatial contiguity matrix. This metric ranges from 0 to 1 and the higher the metric, the more spatially contiguous were the resulting regions.

**References - Appendix S3**

Buhmann, M.D. (2003) Radial basis functions: theory and implementations. Cambridge University Press.

Luxburg, U. (2007) A tutorial on spectral clustering. Statistics and Computing, 17, 395–416.

Tan, P.-N., Steinbach, M. & Kumar, V. (2005) Introduction to data mining, (First Edition). Addison-Wesley Longman Publishing Co., Inc.

Yuan, S., Tan, P.-N., Cheruvelil, K.S., Collins, S.M. & Soranno, P.A. (2015) Constrained spectral clustering for regionalization: Exploring the trade-off between spatial contiguity and landscape homogeneity. Proceedings of the IEEE International Conference on Data Science and Advanced Analytics (DSAA), Paris, France, October 19-21, 2015. DOI: 10.1109/DSAA.2015.7344878.
